# The Complement System in the Setting of Critical Illness—A Narrative Review

**DOI:** 10.3390/biom16040505

**Published:** 2026-03-27

**Authors:** Kleio Ampelakiotou, Ioanna Nikitopoulou, Stelios Kokkoris, Anastasia Kotanidou, Ioanna Dimopoulou, Maria G. Detsika

**Affiliations:** 1 Department of Immunology and Histocompatibility, Evangelismos General Hospital, 10676 Athens, Greece; k.ampelakiotou@gmail.com; 2 GP Livanos and M Simou Laboratories, 1st Department of Critical Care Medicine & Pulmonary Services, Evangelismos Hospital, School of Medicine, National and Kapodistrian University of Athens, 10675 Athens, Greece; joannaniki@gmail.com (I.N.); skokkoris2003@yahoo.gr (S.K.); akotanid@med.uoa.gr (A.K.); idimo@otenet.gr (I.D.)

**Keywords:** complement, intensive care unit (ICU), critical care

## Abstract

The complement system is a key component of innate immunity, known primarily as an immune surveillance mechanism. However, it is also widely known as a modulator of immune responses and inflammation, and its activation has been reported in a wide array of conditions that can lead to admission to the intensive care unit (ICU). Furthermore, various ICU monitoring practices and treatment interventions of the ICU needed to sustain vital organ function may disrupt complement homeostasis. In this review, we will describe in detail the role of the complement system in various critical care settings, with emphasis on major ICU-related conditions such as bacterial and viral sepsis, trauma and burn. Additionally, we will address the potential value of this complex cascade as a prognosis tool and the possible implications for clinical practice as well as its potential as a target for future innovative therapeutic strategies.

## 1. Introduction

A major characteristic of patients admitted to the intensive care unit (ICU) is an increased inflammatory response leading to multiple organ dysfunction syndrome (MODS). This excessive inflammatory response is known to occur regardless of the cause of admission to the ICU [1]. The main causes for ICU admission include sepsis either due to bacterial or viral infections or other conditions not related to infection such as trauma, severe burn, stroke, myocardial infarction, postoperative complications or serious inflammation of a vital organ such as pancreatitis [2].

The complement system is a key mechanism of innate immunity. Although its primary role is to contribute to the detection and elimination of pathogens, under specific circumstances, it is also widely known to promote and sustain inflammation. Admission to the ICU may occur as a result of various medical causes leading to MODS. In most of the aforementioned conditions leading to ICU admission, the complement system has been shown to be activated, thus driving their progression as also stated previously [3]. Moreover, the various treatment interventions provided to ICU patients may contribute further to complement activation resulting in a state of complement overactivation. Such interventions include mechanical ventilation [3,4,5], blood transfusion [6], and extra corporeal membrane oxygenation [3]. For the current review, an extensive literature search was conducted through the PubMed search engine in order to identify recent and older research and review articles. The keywords utilized included the complement system, ICU, and specific ICU-related conditions described in the current review such as bacterial sepsis, viral sepsis, fungal sepsis, trauma and burn. As the focus of the review involves ICU clinical settings, all articles describing in vivo or in vitro basic research studies were excluded and therefore, mechanistic aspects of the relationship between the complement system and ICU settings will not be discussed. Therefore, the aim of the current review is to discuss in detail the various ways by which the complement system plays a role in specific ICU-related conditions and its potential as a tool in clinical practice both in terms of prognosis and as a therapeutic target.

## 2. The Complement System

The complement system is a critical component of innate immunity that is in constant surveillance for invading pathogens, ensuring their removal and destruction while triggering inflammatory signals that reinforce the overall immune response and preserve cellular homeostasis [7].

**Figure 1:**
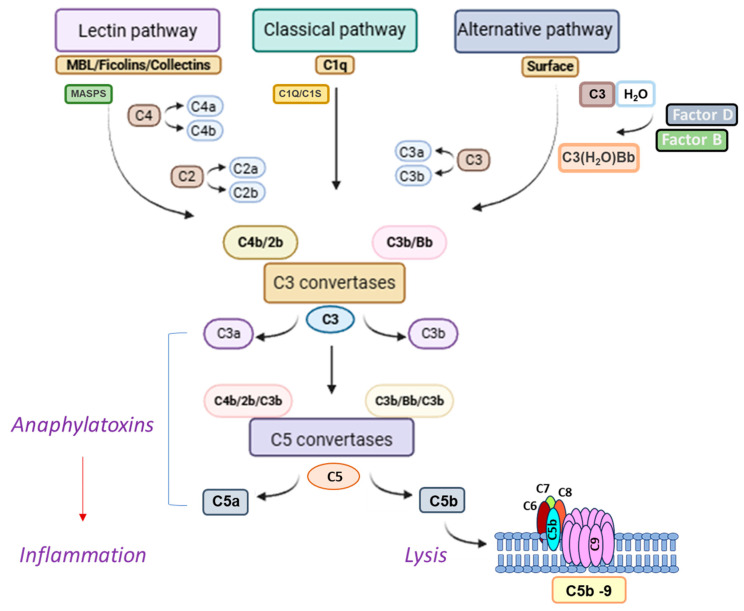
The complement cascade pathways. Initiation of the classical pathway is triggered by binding of C1q to antibody chains thus activating C1s and C1r. Cleavage of activated C2 and C4 molecules into C2a, C2b and C4a, C4b by the activated C1 complex leads to C3 convertase formation after binding of C2b to C4b (C4b2b). Recognition of microbial carbohydrates by ficolins, mannan binding lectin (MBL), or collectins leads to lectin pathway activation. These molecules bind to mannan binding lectin serine peptidases (MASPs) forming a complex. The activated MASPs cleave C2 and C4 into active C2a, C2b and C4a, C4b fragments the C3 convertase (C4b2b) is formed. Alternative pathway activation involves spontaneous C3 activation by C3 hydrolysis. Factor B and Factor D enable the formation of a fluid phase C3 convertase through binding of the hydrolyzed C3 to an active Bb fragment (C3(H_2_O)Bb). C3(H_2_O)Bb cleaves C3 into active C3a and C3b fragments. Binding of active C3b to Bb results in the formation of the alternative pathway C3 convertase (C3bBb). The three pathways join at the C3 step and the activation of the cascade to continue to the C5 step which involves to the formation of the C5 convertase by binding of active C3b to the existing C3 convertases resulting in the formation of active C5 convertases (C4b2bC3b, C3bBbC3b). Circulating C5 is cleaved into active C5a and C5b by the C5 convertases leading to C5b binding to the cell membrane and assembly with C6, C7 and C8 molecules. This further enables polymerization of C9 molecules and formation of C5b-9, also known as membrane attack complex (MAC). Generation of sufficient C5b-9 molecules on the cell membrane leads to cell lysis. The release of C3a and C5a anaphylatoxins and binding to their cellular receptors initiates cellular activation and chemotaxis promoting a strong inflammatory state. Created using BioRender.

This complicated protein network consists of more than thirty plasma and cell membrane proteins which act either as receptors or regulators, most of which are produced by the liver. Activation proceeds through three distinct routes: the classical, the alternative and the lectin pathway. All three pathways lead to a final step, which results in the formation of the membrane attack complex (MAC) that lyses target cells (Figure 1) [7].

The classical pathway is activated when immunoglobulins, IgM or IgG, bind to antigenic epitopes on pathogen surfaces or on soluble immune complexes. C1q mediates C1r autocatalysis, subsequently leading to C1s activation. Active C1s cleave C4 and C2 plasma proteins leading to the generation of C4a anaphylatoxin C4b. Concurrently, cleavage of C2 into C2a and C2b results in the formation of C3 convertase (C4b2b) following binding of C2b to surface-bound C4b [7,8,9]. The lectin pathway is triggered when soluble molecules, known as pattern recognition proteins (PRPs), including mannose binding lectin (MBL), ficolins (ficolin-1, 2, 3), and collectin-1, adhere to distinct carbohydrate motifs displayed on the microbial surface. Each PRP presents multiple carbohydrate recognition domains, enabling high avidity binding to mannose, N-acetylglucosamine, or acetylated sugars frequently present on bacteria. PRPs recruit MBL-associated serine proteases (MASPs). Activated MASP-1 activates MASP-2 leading to C4 and C2 cleavage of C3 convertase (C4b2b) formation [7,8,9].

In contrast to the other two pathways, the alternative pathway sustains low-level activity in the absence of infection. Initiated by the endogenous hydrolysis of the internal thioester bond in circulating C3, C3(H_2_O) is produced, which recruits Factor B (FB). Factor D (FD) will induce proteolysis of FB into Ba and Bb fragments producing the C3(H_2_O)Bb complex which functions as a soluble C3 convertase, cleaving native C3 into C3a and C3b. Binding of C3b to Bb fragment results in the formation of the C3 convertase C3bBb [7,8,9,10]. All three pathways converge on the C3 convertase assembly causing cleavage of C3 into C3a (anaphylatoxin) and C3b (opsonin) active fragments. C5 convertase is produced when a C3b molecule is added to a C3 convertase. C5 convertase cleaves C5 into C5a (anaphylatoxin) and C5b resulting in the binding of C5b to C6, C7, C8, and multiple C9 molecules and the formation of C5b-9, also known as MAC, a perforin-like assembly. MAC creates a transmembrane pore leading to cell lysis. Apart from the direct lysis, the release of potent anaphylatoxin C3a and C5a molecules during complement activation enhance inflammation. When C3a and C5a bind to their receptors C3aR, C5aR1 and C5aR2, a series of inflammatory events are initiated, leading to neutrophil, eosinophil, monocyte and T lymphocyte recruitment and the activation of phagocytic and endothelial cells [7,9,11].

### Complement Regulation

**Figure 2:**
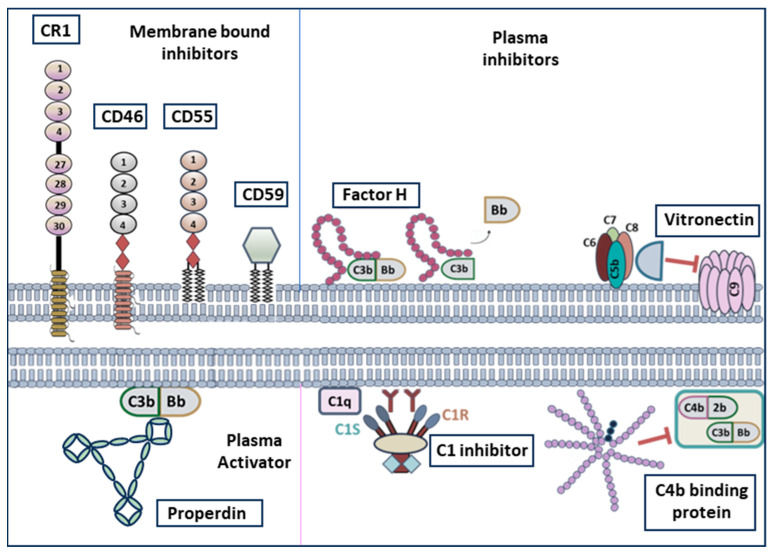
Complement regulation by activating and inhibiting protein molecules. Schematic representation depicting negative complement regulatory proteins (CRegPs), membrane bound (CR1, CD46, CD55, CD59) and soluble (Factor H, Vitronectin, C1 inhibitor, C4b binding protein), and positive regulator properdin. (Adapted by Detsika M.G., et.al., 2024 [9]).

Complement regulatory proteins (CRegPs) form an essential barrier against inadvertent host damage by constraining complement activation ensuring that complement effectors act exclusively upon designated targets. CRegPs are either membrane bound or soluble and tend to share similar structural and functional characteristics (Figure 2 and Table 1) and may act at either the C3 or C5 steps of the cascade, or both. Membrane bound CRegPs include CD55, also known as decay accelerating factor (DAF), [12,13], CD46, also known as membrane cofactor protein (MCP), [14,15], CD59 [16,17] and CR1 [14]. Fluid phase CRegPs include C4b binding protein [18,19,20,21], C1 inhibitor [22,23], Factor H (FH) [24,25,26,27] and vitronectin [28].

Another protein which is known to regulate the activation of the complement system is Properdin [30]. Properdin is a plasma glycoprotein and the only known positive regulator of the complement system activating exclusively on the alternative pathway [29,31].

## 3. Bacterial Sepsis

Data from clinical studies involving sepsis patient samples have indicated that complement activation is initiated differently by Gram-positive and Gram-negative bacteria that cause bacterial septicemia [31]. In patients with confirmed Gram-positive bacteria-induced sepsis, a significant consumption of C1q, but not MBL, has been reported in the acute phase of the disease, while the opposite was observed in sepsis caused by Gram-negative bacteria. C1q can bind directly to bacterial surfaces or to immune complexes formed by bacterial antigens and antibodies. In the case of Gram-negative pathogens, the activation of the complement is induced in sepsis through the binding of MBL predominantly to lipopolysaccharide (LPS) [31].

Various studies have also recently demonstrated that serum C1q levels are decreased in sepsis and related to sepsis severity, organ damage and coagulation function. Serum C1q may also serve as a potential useful marker in sepsis prognosis [32]. Specifically, the expression level of C1q in neutrophils was reported as a distinct characteristic of a subpopulation of sepsis patients who have severe immune dysregulation and higher mortality [33]. Therefore, the heterogeneous upregulation of C1q during sepsis may correlate with enhanced neutrophil reactivity. This finding could help in understanding the mechanisms of the microbiome–neutrophil interaction in homeostasis, as well as potential connection with C1q production in sepsis.

Another study suggested that C3 and C4 concentrations on admission in patients with Gram-negative bacteremia may help to classify patients with increased risk for septic shock and mortality [34]. The study involved patients with *Staphylococcus aureus* and Gram-negative bacteremia as compared to non-bacteremic hospitalized patients and community controls and investigated possible a association between complement levels and patient clinical outcome. The findings suggested that complement profiles among the patients differ. C5a levels were significantly higher in the bacteremic groups as compared to the non-infected and community control groups and were also significantly higher in the *Staphylococcus aureus* cohort as compared to Gram-negative bacteremia.

C3d lung deposition has been verified in ICU patients, regardless of the degree of alveolar damage and acute respiratory distress syndrome (ARDS) diagnosis [35]. Complement activation in septic patients receiving antibiotics and high-dose corticosteroids was previously studied, revealing that C1 inhibitor, and C3, C4, and C5 in plasma were low before treatment but increased one week later. On the other hand, levels of anaphylatoxins C3a and C5a were elevated on admission, and after successful treatment decreased to normal [36]. Importantly, plasma C3a is associated with the clinical outcome of sepsis patients, as C3a levels of non-survivors were significantly higher than those of survivors [37].

Other studies have also demonstrated that monitoring the complement system can help to predict outcomes for sepsis patients. The time courses of serum complement components and the severity of sepsis were compared in survivor and non-survivor septic patients at several points after the diagnosis. C3 and C4 levels were significantly lower in the non-surviving than in the surviving group, while the levels of C3a and C4a were significantly higher in the non-surviving than in the surviving group [38].

Signaling through anaphylatoxin C3a can result in disruption to bacterial membranes, independent of opsonophagocytosis [39]. However, abnormal anaphylatoxin signaling can lead to excessive tissue injury and bacterial immune evasion. In community-acquired pneumonia, increased circulating levels of C3a may serve as an early diagnostic biomarker since they correlated with the severity of the disease [40]. Inhibiting the complement system reduces *Streptococcus pneumoniae*-elevated secretion of inflammatory cytokines in pulmonary epithelial cells, pointing to a role for C3a in the pathophysiology of community-acquired pneumonia. It is worth mentioning a recent analysis of 36 studies including 6330 sepsis patients that revealed lower C3 and C4 levels and higher C4a in sepsis non-survivors [41]. This review and meta-analytic study involved the MIMIC-IV clinical dataset [42] and three independent proteomic studies. As lower circulating levels of classical complement proteins is a known feature of sepsis non-survivors, these findings suggest that complement pathway status profiling may serve as a potential biomarker tool for risk stratification and a target for immunotherapy.

Properdin levels in sepsis patients upon ICU admission were decreased in comparison to healthy controls, but increased after sepsis recovery, and were lower in non-survivors compared to survivors [43]. The major role of the alternative pathway in sepsis was recently depicted in sepsis patients, where complement activation induced thrombocytopenia, increased mortality and enhanced sepsis severity, measured as APACHE2 and SOFA scores [44]. MBL is a C-type lectin with a crucial role in the host defense system against microbial infection. MBL recognizes the patterns of glycans presented by microorganisms and then opsonizes antigens and activates the lectin pathway of the complement system [45]. Human MBL is encoded by a single gene (MBL2), where six single-nucleotide polymorphisms (SNPs) can be found and can affect the structure and levels of the MBL protein. MBL deficiency caused by MBL2 gene polymorphisms is linked with increased risk and severity of several infections [46,47]. Further exploration revealed that low MBL-producing genotypes and low MBL concentrations are significant risk factors for persistent *Staphylococcus aureus* bacteremia [48].

Various clinical studies have previously demonstrated a strong complement activation in patients with severe sepsis, and the production of anaphylatoxins especially was found to be associated with a fatal outcome [49]. Increased plasma levels of C5a during sepsis returned to normal after successful treatment [36].

In patients suffering from bacteremia, neutrophils show changes indicative of nonspecific deactivation and receptor assays reveal loss of C5a-C5aR interaction [50]. Meanwhile, determination of C5aR1 and C5aR2 neutrophil expression can be helpful in early diagnosis of sepsis [51].

Of note, pathogens like *Haemophilus influenzae* can evade host immune attack by producing complement inhibitory proteins that reduce production of C5a and neutrophil activation in the airway, allowing for chronic colonization [52].

### Complement Regulatory Proteins and Bacterial Sepsis in ICU

Neutrophils express complement receptor-1 (CR1 or CD35) that interacts with the C3b-opsonized particles and mediates pathogen recognition by phagocytic cells. It was observed that the staphylococcal extracellular complement binding protein prevents recognition of C3b-opsonized bacteria by neutrophil CR1, resulting in limited phagocytosis and immune evasion of *Staphylococcus aureus* [53].

Recently, it was demonstrated that at ICU admission there was a rapid decrease in CR1 membrane expression of red blood cells from septic patients [54]. Surface CR1 protein protects red blood cells from the enhanced activation of the complement system during sepsis; thus, the observed significant decrease at ICU admission in CD35 expression persisting at day 3–5 points out the evolution of the regulatory protein in sepsis.

C1 esterase inhibitor (C1-INH) regulates the complement pathway via inactivation of C1r, C1s, and mannose-binding protease-associated serine protease 2. In addition, C1-INH modulates coagulation leukocyte adherence. In the case of C1-INH deficiency, released bradykinin can mediate increased vascular permeability. A recombinant strain of Streptococcus pyogenes was shown to interrupt the human complement system via degradation of C1-INH, thus enabling immune evasion [55]. More specifically, a major extracellular cysteine protease, known as SpeB of *Staphylococcus pyogenes*, was shown to possess cleaving activity for C1-INH in human serum, causing structural change and protein dysfunction. Therefore, it is considered that *Staphylococcus pyogenes* escapes the human complement system by the cleaving activity of SpeB, which allows the pathogen to survive with an advantage in serum containing C1-INH.

CD46 is involved in factor I-mediated inactivation of C3b and C4b. In sepsis, CD46 participates in immune response acceleration upon meningococcal infection through regulation of macrophage polarization and survival [56].

A recent study identified a role for FH in the preservation of the alternative complement pathway activation which was linked to survival. The study used clinical samples from two large cohorts, the ARDSnet Lisofylline and Respiratory Management of Acute Lung Injury (LARMA) trial with 218 patients and the ARDSnet LARMA and Statins for Acutely Injured Lungs from Sepsis (SAILS) trial with 224 patients, in order to measure total alternative pathway activation and FB and FH levels in each of the studies respectively. Subsequently, they performed a meta-analysis on LARMA and the X Acute Lung Injury Registry and Biospecimen Repository (ALIR) observational registry and found an association of AH50 levels greater than the median with reduced mortality. Furthermore, in a subset of these patients, they detected FH deficiency as measured by lower FB and C3 levels and Ba:B and C3a:C3 ratios. This deficiency was associated with increased mortality [57].

Another recent study reported that SNPs in genes encoding alternative complement pathway components such as FH were identified to have potential clinical relevance and may contribute to individual susceptibility to bacteremia and sepsis [58].

## 4. Viral Sepsis—Coronavirus Disease-2019 (COVID-19)

In the recently emerged COVID-19 caused by SARS-CoV-2 outbreak, which quickly evolved into a pandemic, the role of the complement system was investigated in depth. It is now widely known that mortality due to COVID-19-associated respiratory failure was associated with specific vascular features of the lung, including severe endothelial injury associated with the presence of disrupted cell membranes and intracellular viruses, as well as extensive thrombosis with pulmonary vessel microangiopathy [59]. A number of studies revealed increased complement fragment levels in critically-ill COVID-19 patients demonstrating a state of complement overactivation in these patients [4,60,61,62,63], with a possible contribution to their highly inflammatory state. C3a, C5a, C5b-9 and C4d were found to be significantly increased in critically-ill patients and correlated with specific disease characteristics and antiviral antibodies. The above findings strongly suggest the potential of these molecules as possible disease progression markers [4,59,60,61,62,63]. Another study identified an association between increased plasma C5a levels with COVID-19 severity, and increased blood and myeloid cell C5aR expression [64]. Recently, de Andrande et.al reported significantly higher levels of plasma C2, C5a, FB and FD in patients requiring ICU admission [65]. Another study reported the detection of dysregulated complement activation in lung tissue samples of patients with non-resolved COVID-19 [66]. The study identified increased mRNA expression of C1q, C1r, C1s, C3 and C5a, as well as of the anaphylatoxin receptor C5aR1, in NR-COVID-19 lung tissues. Furthermore, the same study detected decreased expression of all CRegPs at the mRNA level but not at the protein level apart from the CD55 protein which was detected at lower levels in NR-COVID-19 lung tissues compared to controls [66]. The decrease in CRegPs at the tissue level contrasted with findings regarding CRegPs expression in the peripheral blood mononuclear cells (PBMCs) of COVID-19 patients. Using an omics approach, Detsika et.al., investigated the expression of CRegPs in PBMC subpopulations of COVID-19 patients with critical illness and identified an increased expression of CD55 in T cells of critically-ill COVID-19 patients [67]. This finding was further linked to the suppression of type-I interferon (IFN) responses in these patients and therefore indicated a role of CD55 in COVID-19 host immune response and progression. The role of C3 was also highlighted in another ‘omics’ study which combined a single-cell RNA sequencing and CyTOF approach. The study detected increased generation of C3a in critically-ill COVID-19 patients and reported that a C3-rich environment in these patients induced the activation of highly cytotoxic CD16^+^ T cells which was associated with poor prognosis and a fatal outcome [68]. A potential involvement of complement in the platelet/NET/thrombin axis has also been reported in critically-ill COVID-19 patients [69]. The central role of the complement system in net-driven immunothrombosis in COVID-19 patients was shown in a study, which demonstrated high tissue factor expression in neutrophils from COVID-19 patients. This was disrupted by the use of a C3 inhibitor while C5aR1 blocking or netosis and thrombin inhibition in neutrophils from COVID-19 subjects eliminated platelet-mediated NET-driven thrombogenicity [69].

## 5. ICU Admission Due to Other Pathogens

The complement response is a necessary part of host immunity against all pathogens including fungi and parasites [70,71]. Although over the past few decades, major advances in contemporary critical care have significantly improved patient survival rates, these same advances have also been reported to contribute to a growing incidence of opportunistic fungal infections [72]. In 2007, the results of the EPIC II study including 1265 ICUs in 75 countries revealed that 19% of pathogens isolated in ICU patients were fungi with the most frequently encountered invasive fungal infection being candidaemia, followed by *Aspergillus* species infection [73]. Fungi may cause a wide spectrum of diseases, but only a limited number are pathogenic to humans, with *Candida*, *Aspergillus*, and *Cryptococcus* being responsible for most invasive infections frequently observed in the ICU [74].

Recent studies have also demonstrated the crucial role of the complement system in antifungal host defense. A previous study reported that the lack of complement effector proteins C3 and C5 result in marked susceptibility to systemic fungal infection [75] and that the absence of C3 did not affect early inflammatory responses but delayed fungal clearance. Thus, C3 seems to play an essential role in the control of opportunistic fungal infections and might potentially contribute to mortality [76]. Another study demonstrated the essential role of C5a-C5aR1 signaling in phagocytes in host immune capacity against *Candida* and for effective fungal clearance and host survival. This mechanism is based on locally produced C5 within *Candida*-infected tissue by phagocytes, denoting that C5 is an important mediator during systemic fungal infection. Cryptococcosis is another serious, often fatal, fungal infection caused primarily by *Cryptococcus neoformans* and *Cryptococcus gattii* that mainly affects immunocompromised individuals. The activation of the complement system against cryptococcal infection has also been reported by various studies [77]. The lack of C5 has been shown to result in worse progression of cryptococcal infection [78] while in patients with cryptococcal fungemia, low levels of C3 and FB have been observed [79]. Furthermore, a study involving complement component profiling in human cerebrospinal fluid and plasma samples from individuals with Cryptococcal meningitis revealed that levels of C1q, FB, MBL, C5b-9 and FH were increased compared with controls, denoting increased activity of the complement system [80]. Mucormycetes form a heterogeneous group of fungi, some species of which can act as opportunistic pathogens and cause a disease called mucormycosis and the complement was shown to play a key role in fighting mucormycetes off [81]. The complement system plays a critical role in defense against invading pathogens, but obligatory intracellular parasites have developed highly advanced mechanisms to modulate or suppress this humoral first line of defense, enhancing their ability to establish and sustain infection [82]. *Plasmodium falciparum* infection causes the majority of deaths due to severe and cerebral malaria. In human malaria infections, increased complement activation has been detected [83]. Specifically, the roles of C5a and C5aR were experimentally investigated and shown to contribute to the pathogenesis of cerebral malaria through decreased inflammation, decreased endothelial activation, and improved blood–brain barrier integrity [84].

Furthermore, regarding the neurotropic parasite *Toxoplasma gondii*, the infection with which results in significant brain alterations, the involvement of the complement pathway has been examined in the context of the initial immune response [85]. Another study investigating the expression and localization of cerebral C1q in chronic *Toxoplasma gondii* infection showed that C1q activation occurs during chronic toxoplasmosis as part of the immune response [86]. Moreover, *Toxoplasma* infection was found to increase the mRNA levels of Factor B and C5a receptors [87,88].

## 6. Trauma

Severe trauma triggers a rapid initiation of the coagulation cascade, and this hemostatic response directly intersects with the complement system. Studies have shown that key coagulation enzymes, including thrombin [89,90,91], plasmin [92], and factor Xa [93], are capable of cleaving C3 and C5, generating the potent anaphylatoxins C3a and C5a, which amplify inflammation and leukocyte recruitment. In addition, trauma-associated contact activation can stimulate factor XII, which in turn activates C1 esterase and initiates the classical complement pathway [94]. Complement activation also shapes platelet behavior, as thrombocytes express several complement receptors that can be engaged during systemic inflammation. Activation of these receptors influences platelet aggregation affecting their overall integration into the coagulation process [95].

Several studies have confirmed that polytrauma triggers a rapid and pronounced activation of the complement system, reflected by elevated C3a/C3 ratios as well as increased levels of C5a and the terminal complement complex C5b-9. Specifically, one study involving 40 polytraumatized patients reported markedly elevated C3a levels for up to 10 days following injury, negatively correlating with Glasgow Coma Scale score, a finding which indicated sustained complement activation during the post traumatic phase. The study also showed that patients who ultimately died from multiple trauma exhibited higher concentrations of serum C3a during their stay in the emergency department, highlighting the potential prognostic value of C3a as a biomarker for trauma-related mortality [96].

Another study involving 108 polytrauma patients reported a positive correlation between C3 activation at admission with Trauma Score, indicating that early complement activation reflects initial injury severity. Moreover, the highest measured C3bc levels were significantly associated with Injury Severity Score (ISS), further linking complement activation to the extent of tissue damage. Consistent with this pattern, ISS was also positively correlated with both admission and peak concentrations of the terminal complement complex C5b-9. Notably, patients who progressed to sepsis exhibited elevated levels of both C3 activation products and C5b-9, suggesting that excessive or dysregulated complement activation may contribute to complications following severe trauma [97].

Complement component C5a showed a similar pattern of dysregulation. Serum C5a levels have been reported to remain elevated throughout the duration of the observation with a pronounced peak at approximately 120 h after injury, suggesting an ongoing complement activation. A positive correlation between C5a and brain injury was observed while C5a levels served as an indicator of patient survival, with higher concentrations associated with poorer outcomes [98].

In the same study, an increase in the terminal complement complex, C5b-9, was determined in the samples that were collected upon arrival to the ER followed by a decline of the concentrations comparable to those of the healthy individuals between four and 24 h after trauma. Interestingly, a further elevation in C5b 9 levels was observed approximately five days after the traumatic event [98].

Activation of the complement system was also reported in another study which measured C5b-9 levels early after trauma [99]. The study revealed elevated C5b-9 levels as early as 20 min following admission and provided evidence for a central role of the alternative pathway. Furthermore, the study also reported that in patients with low C3a levels, there was an association between increased C5b-9 levels and thrombin thus indicating a C3-independent mechanism of complement activation by thrombin in these patients [99].

Finally, another study reported that trauma patients with ARDS exhibited markedly reduced levels of C4, C1 inhibitor, FH, and Factor I during the first 24 h after injury compared with healthy controls. These complement components gradually returned to baseline between days four and 14. In contrast, C3 concentrations also decreased during the initial 48 h but began to rise from day four onward. Despite this upward trend, C3 levels remained significantly lower in patients with ARDS throughout the entire 14-day observation period, indicating a sustained impairment of complement activity [99].

## 7. Burn

Activation of the complement system has been detected in patients with severe burns in a study which reported decreased levels of C3 in patients with ≥20% burns compared to healthy controls as well as compared to patients with ≤20% burns. The same study reported lower C3 levels in non-survivors compared to survivors and suggested the prognostic value of complement markers in these patients [100]. Another study involving patients with severe thermal injury also reported C3 decreased levels indicative of complement consumption in the initial study time point [101] accompanied by an increase in the following time points. The study reported that recovery of C3, CH50 and AH50 levels during the early days of treatment correlated with survival and better prognosis [101]. Furthermore, increased levels of C3a and C5a were measured in another study on severe thermal injury patients which monitored C3a and C5a levels over a course of three weeks [102]. The study reported a peak of C3a levels during the second week while C5a levels peaked earlier; meanwhile, no correlations were observed regarding outcome or sepsis development [102]. Another study determined an increase in FB and thus the activation of the alternative pathway in these patients [103].

## 8. Clinical Implications—ICU Prognosis and Potential Therapeutic Targets

The various studies described in the above sections demonstrate how the complement system is activated in various ICU settings and a list of these studies is included in this review in Table 2. The activation of the complement system in patients admitted to the ICU renders it a potential candidate for use in clinical practice, specifically for disease progression prognosis and outcomes. Indeed, the prediction of outcomes such as mortality has been demonstrated in various ICU conditions such as bacterial [41] and viral sepsis, particularly in COVID-19 [4], as well as in trauma and burn [100]. All aforementioned studies demonstrated a strong prognostic value of complement components in ICU settings. Specifically, a strong correlation with mortality was shown for anaphylatoxins C3a (area under the curve (AUC) =0.740, *p* < 0.0001) and C5a (r = 0.5366, *p* = 0.0047) [60] as well as for C5b-9 (AUC = 0.603) [63], while low properdin levels were found to correlate with need for intubation (AUC = 0.82, *p* = 0.002). Extensive analysis on the correlation of complement components levels with COVID-19 progression and outcome strongly suggest their future potential in clinical practice [104]. Although the measurement of specific complement components such as complement fragments and anaphylatoxins may be laborious and fairly expensive, given the precision of the outcome prediction by these molecules, they might hold a strong value as biomarkers in ICU clinical practice.

The involvement of the complement system in various conditions also renders it a potential target for the development of novel therapies. The three different complement activation pathways and the numerous protein molecules which comprise this complex cascade offer a wide spectrum of potential target molecules at the different steps of the cascade. The interest of pharmaceutical companies for the development of complement targeted therapeutics has risen in recent years and there are currently multiple different molecules and agents under development for various diseases, including complement-mediated nephropathies as well as cancer. The administration of Eculizumab, a well-known C5a inhibitor which has already been approved for the treatment of paroxysmal nocturnal hemoglobinuria, has been reported as a potential therapeutic option in complement-mediated thrombotic microangiopathy which developed secondary to sepsis. This case report study demonstrated the effectiveness of administration of Eculizumab to a sepsis patient who developed thrombotic microangiopathy accompanied by disseminated intravascular coagulation [102]. Complement-related therapeutic strategies were also employed in the ICU setting during the COVID-19 pandemic. Numerous complement-targeted agents were employed in clinical trials in the battle against COVID-19, especially in critically-ill patients, including C5 inhibitors, C3 inhibitors, C1 inhibitors, and FD inhibitors, with variable effects [104,105]. Some of these trials showed remarkably promising results such as the administration of vilobelimad, an anti-C5a agent [106], while others showed marginally beneficial effects [105,107]. Administration of eculizumab resulted in the improvement of ARDS by reducing the need for oxygenation and inflammation, as well as hospitalization duration when used alone [104,105] or in combination with the JAK1-2 inhibitor ruxolitinib [107]. The use of vilobelimab, another C5a inhibitor, in COVID-19 patients requiring mechanical ventilation improved survival [105] and was granted FDA approval for emergency use in COVID-19 hospitalized adults [106]. Narsoplimab, a lectin pathway inhibitor, was also tested in COVID-19 patients with severe disease. When administered to patients requiring continuous positive airway pressure (CPAP) or intubation, this MASP-2 inhibitor resulted in a decrease in inflammatory markers and a reduction in mortality [108]. Another study described administration of a C3 inhibitor, namely AMY-101, in COVID-19 patients with severe ARDS which reduced inflammation and ameliorated the syndrome [107,109], albeit without statistical significance in larger trials [109,110].

## 9. Conclusions

The complement system remains an important mechanism that may drive the progression of various conditions presented within the ICU. Understanding the role of this mechanism is of crucial importance as it may enable the discovery of novel therapeutic targets and open new therapeutic avenues. Unraveling the complex role of this cascade in the various ICU conditions is essential in order to achieve the necessary fine-tuning of the immune and inflammatory response by administration of these potential novel therapeutics and to improve clinical practice and disease outcomes. Furthermore, a deeper understanding of the complex pathophysiological processes underlying critical illness may assist the choice of novel therapeutic strategies with a broad clinical benefit in order to improve clinical practice in the ICU.

## Figures and Tables

**Table 1:** Structural and functional characteristics of complement regulatory proteins in humans. Short consensus repeats, SCRs; thrombospondin type I repeats, TSR.

Membrane Bound	Structure	Function	Reference
CR1	Attachment via a transmembrane domain- 30 SCRs	Binds and deactivates C1r and C1s	Kim D.D et al., 2006 [14]
CD55 (Decay accelerating factor, DAF)	Attachment via a gpi anchor, 4 SCRs, serine–threonine-rich structure	Accelerates decay of C3 and C5 convertases	Lublin D.M. et al., 1989 [12]
CD46 (Membrane cofactor protein, MCP)	Attachment via transmembrane domain- 4 SCRs, serine–threonine-rich structure	Acts as a cofactor for factor I-mediated degradation of C3b and C4b	Kim D.D et al., 2006 [14]
CD59	Attachment via a gpi anchor, extracellular domain with disulphide bond-linked amino acids	Prevents C5a binding and C5b-9 assembly	Podack E.R. et al., 1984 [16]
**Plasma/circulating proteins**			
C1 inhibitor	Serine protease inhibitor (serpin) consists of 2 domains, a C-terminal serpin domain and a N-terminal non-serpin domain	Prevents C1s and C1r activation by binding to C1q	Davis A.E et al., 2010 [22]
C4b binding protein	7 identical α-chains and one β-chain α-chain; 8 SCRs and a C-terminal oligomerization domain, β-chain; 3 SCRs and a C-terminal oligomerization domain	Facilitates C3 convertases dissociation	Rodriguez de Cordoba S. et al., 1991 [19]
Factor H	20 SCRs	Binds the alternative pathway C3 convertase and facilitates its dissociation	Wu J. et al., 2009 [27]
Vitronectin	75 kDa single chain, or 2 chains: (65 and 10 kDa) linked by a disulphide bond	Prevents C5b-9 assembly	Milis L. et al., 1993 [28]
Properdin (positive regulator)	Monomeric subunits structures, each containing 6 full TSRs	Stabilizes alternative pathway C3 convertase and promotes C3b and FB association for C3 convertases formation	Alcorlo M. et al., 2013 [29]

**Table 2:** List of references reporting modulation of complement activation in ICU.

	Component Altered	Complement Pathway Modulation	Reference
**Bacterial sepsis**			
	C1q	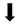	Li H. et al., 2021 [32]
	C3	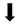	Eichenberger E. M. et al., 2020 [34]
	C3a	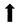	Bengtson A. et al., 1988 [36]
	C4	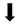	Eichenberger E. M. et al., 2020 [34]
	Properdin	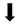	Stover C. M. et al., 2015 [43]
	MBL	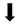	Chong Y. P. et al., 2014 [48]
	C5a	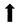	Bengtson A. et al., 1988 [36]
	C5aR	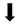	Furebring M. et al., 2002 [51]
**COVID-19**			
	C3a	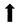	Alosaimi B. et al., 2021 [60]
	C5a	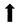	Alosaimi B. et al., 2021 [60]
	C4d	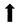	Sinkovitz G. et al., 2021 [63]
	sC5b9	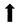	Henry B.M. et al., 2021 [61]
	C5aR	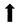	Carvelli J. et al., 2020 [64]
	CD55	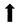	Detsika M.G. et al., 2025 [67]
	C2, C5a, FB, FD	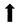	de Andrande. V. L. et al., 2025 [65]
**Trauma**			
	sC5b-9	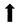	Fosse E. et al., 1998 [97]
	C3bc	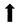	Fosse E. et al., 1998 [97]
	C3	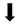	Zillow G. et al., 1992 [99]
	C3a	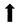	Mannes M. et al., 2021 [96]
	C5a	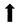	Burk A. M. et al., 2012 [98]
	C4	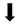	Zillow G. et al., 1992 [99]
	FH	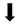	Zillow G. et al., 1992 [99]
	Factor I	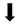	Zillow G. et al., 1992 [99]
**Burn**			
	C3	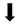	Modi S. et al., 2014 [100]
	C3a	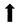	Moran K. T. et al., 1987 [102]
	C5a	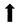	Moran K. T. et al., 1987 [102]
	FB	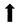	Wan K. C. et al., 1998 [103]

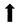
: increase, 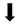: decrease.

## Data Availability

No new data were created or analyzed in this study.

## Abbreviations

The following abbreviations are used in this manuscript:

ICU	intensive care unit
MODS	multiple organ dysfunction syndrome
MAC	membrane attack complex
PRPs	pattern recognition proteins
MBL	mannose binding lectin
MASPs	MBL-associated serine proteases
FB	factor B
FD	factor D
FH	factor H
CRegPs	complement regulatory proteins
DAF	decay accelerating factor
MCP	membrane cofactor protein
LPS	lipopolysaccharide
CR1	complement receptor-1
C1-INH	C1 esterase inhibitor
LARMA	Respiratory Management of Acute Lung Injury
SAILS	Statins for Acutely Injured Lungs from Sepsis
ALIR	Acute Lung Injury Registry and Biospecimen Repository
COVID-19	Coronavirus disease
PBMCs	peripheral blood mononuclear cells
IFN	interferon
ARDS	acute respiratory distress syndrome
